# Assessment of Radiation Exposure in a Nuclear Medicine Department during ^99m^Tc-MDP Bone Scintigraphy

**DOI:** 10.3390/toxics11100814

**Published:** 2023-09-26

**Authors:** Suphalak Khamruang Marshall, Piyatida Prom-on, Siriluck Sangkue, Wasinee Thiangsook

**Affiliations:** 1 Department of Radiology, Faculty of Medicine, Prince of Songkla University, Songkhla 90110, Thailand; 2 Molecular Imaging and Cyclotron Center, Division of Nuclear Medicine, Department of Radiology, Faculty of Medicine, Prince of Songkla University, Songkhla 90110, Thailand; 3 Department of Physics, Faculty of Science and Technology, Suratthani Rajabhat University, Suratthani 84100, Thailand

**Keywords:** bone scan, occupational radiation exposure, low-dose radiation, radiation protection, SPECT, nuclear medicine, radiation exposure, dose rate, radiology, ^99m^Tc

## Abstract

This study measured ^99m^Tc-MDP bone scintigraphy radiation risks, as low-dose radiation exposure is a growing concern. Dosimeter measurements were taken at four positions (left lateral, right lateral, anterior, and posterior) around the patients at 30, 60, 100, and 200 cm at 0, 1.5, and 3 h. The highest dose rates were recorded from 51% of the patients, who emitted ≥ 25 µSv/h up to 49.00 µSv/h at the posterior location at a distance of 30 cm. Additionally, at the anterior location at a distance of 30 cm, 42% of patients emitted ≥ 25 µSv/h up to 38.00 µSv/h. Furthermore, at 1.5 h after the tracer injection, 7% of the dose rates exceeded 25 µSv/h. There was a significant reduction in mean dose rates for all positions as distance and time increased (*p*-value < 0.05). As a result, radiation levels decreased with increased distance and time as a result of radiation decay, biological clearance, and distance from the source. In addition, increasing the distance from the patient for all positions reduced the radiation dose, as was substantiated via exponential regression analysis. Additionally, after completing the bone scintigraphy, the patients’ dose rates on discharge were within the current guidelines, and the mean radiation doses from ^99m^Tc-MDP were below occupational limits. Thus, medical staff received less radiation than the recommended 25 μSv/h. On discharge and release to public areas, the patients’ mean dose rates were as follows: 1.13 µSv/h for the left lateral position, 1.04 µSv/h for the right lateral, 1.39 µSv/h for the anterior, and 1.46 µSv/h for the posterior. This confirms that if an individual was continuously present in an unrestricted area, the dose from external sources would not exceed 20 µSv/h. Furthermore, the patients’ radiation doses were below the public exposure limit on discharge.

## 1. Introduction

Worldwide, ionizing radiation for medical diagnostics and therapeutics has expanded quickly and become a substantial cause of potential radiation exposure to patients and medical staff [1]. Therefore, there is an increasing obligation to minimize radiation risks such as tissue damage and cancer [2]. In fact, a study by Rehani et al. established that 1.33% of patients undergoing computerized tomography (CT) examinations had received a cumulative effective dose (CED) of radiation ≥ 100 mSv [3]. The International Commission on Radiological Protection (ICRP) Publication 103 sets a limitation of 1 mSv/y for members of the public [4] and 20 mSv/y for nuclear medicine staff (averaged over 5 consecutive years) and a maximum of 50 mSv/y in any single year [5]. Concern regarding low-dose radiation safety is growing. Khamtuikrua, Chaowanan, and Sirilak Suksompong determined that medical staff have an acceptable perception of but insufficient knowledge about radiation danger and protection, and there is a need for ongoing training in nuclear safety [6]. Furthermore, radiation safety and dosage optimization at low doses may need to be reconsidered because it is believed that radiation damage may have been grossly underestimated.

Bone scintigraphy examinations are one of the most commonly performed diagnostic nuclear medicine imaging procedures, and provide whole-body bone surveys [7]. In nuclear medicine diagnostic imaging, the technetium-99m (^99m^Tc) radiotracer is the most frequently used radionuclide because of its physical characteristics of a short physical half-life of 6.04 h, a 140 keV monoenergetic gamma emission, and a 90% external photon yield. The gamma radiation emitted from ^99m^Tc produces non-negligible irradiation to adjacent tissue, as a substantial fraction of the gamma rays exit the patient’s body with little interaction. The 6.04 h half-life and quick elimination of the radiopharmaceutical provide sufficient time for diagnostic imaging while minimizing the patient’s radiation exposure. For this reason, ^99m^Tc is regularly used to assess bone formation associated with benign and malignant diseases. ^99m^Tc is complexed to create a radiotracer with either methylene di-phosphonate (MDP) to form ^99m^Tc-MDP or hydroxydiphosphonate (HDP) to form ^99m^Tc-HDP. After administration, within 3–4 h, approximately 50–60% of diphosphonate tracer chemisorption uptake to the bone occurs [8]. In children, bone absorption is localized in the metaphyseal growth zones, which can lead to higher-than-normal dosage absorption in these locations. Furthermore, the differences in biodistribution, dosing, clearance mechanisms, and radiation sensitivity between pediatric and adult patients are crucial for optimizing the use of ^99m^Tc in pediatric imaging to ensure accuracy and safety [9,10]. Different pediatric renal clearance rates may influence the speed and extent of ^99m^Tc elimination. Therefore, monitoring the extraction process to ensure proper renal function is critical to managing radiation exposure and optimizing the quality of the scan [11,12]. In addition, bone uptake is enhanced by increased blood flow and increased osteoblastic activity. The procedure for bone scintigraphy begins with an intravenous injection of ^99m^Tc-MDP or ^99m^Tc-HDP. The ^99m^Tc activity level for adults is 300–740 MBq with an effective dose of 2.9–4.2 mSv. Moreover, ICRP Publication 53 contains ^99m^Tc-labeled phosphates and phosphonates with their estimated organ-absorbed radiation doses [13]. In most cases, the activity that is administered to adults is within the range of 8 to 10 MBq/kg. Furthermore, the models from ICRP 128 claim that a ^99m^Tc-MDP 740 MBq injection to an adult would deliver an effective dose of 4.2 mSv [14]. Additionally, the effective dose for the public is generally less than 1 mSv. To put this into perspective, the average annual background radiation exposure from natural sources is approximately 1–3 mSv per year. Therefore, the exposure from a ^99m^Tc-MDP bone scan is much lower than typical background radiation exposure over the course of a year. It is important to emphasize that medical professionals take precautions to minimize radiation exposure during imaging procedures and follow strict guidelines and safety protocols to ensure the safety of both patients and the public [15,16,17].

The main objective of this study was to ascertain the radiation dose rate emitted at various positions from patients undergoing bone scintigraphy, to establish any potential risk to medical staff and, additionally, members of the public after patient discharge.

## 2. Materials and Methods

### 2.1. Materials and Methodology

**Figure 1:**
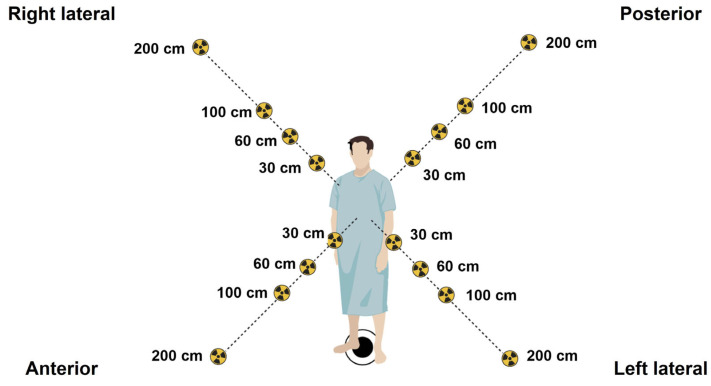
Radiation intensities were measured at chest level from 4 positions around the body (left lateral, right lateral, anterior, and posterior) at distances of 30, 60, 100, and 200 cm postinjection, and at time points of 0 h (injection time), 1.5 h (waiting time), and 3 h (scanning/discharge time) to determine the mean dose rate (µSv/h) emitted from 100 patients undergoing ^99m^Tc-MDP bone scintigraphy.

This study was performed at the Division of Nuclear Medicine, Department of Radiology, Songklanagarind Hospital, Thailand, where 100 adult patients aged 18–86 years old underwent bone scintigraphy between July 2021 and December 2021. They were each administered a 740 MBq (20 mCi) activity of ^99m^Tc-MDP and imaged 2–4 h after intravenous injection with a Philips dual-head planar γ-camera. In our center, a standardized ^99m^Tc-MDP activity is administered to each patient, rather than adjusting the activity based on the patient’s weight. This is based on achieving consistency in imaging protocols, resulting in a more efficient workflow, image quality, and radiation dose. However, in pediatrics, adjustments are based on patient weight or body surface area to optimize the balance between image quality and radiation dose. All patients undergoing scintigraphy were monitored for mean radiation over a period of 3 h using a Fluke 481 radiation survey meter to detect deep-dose (gamma) radioactivity (Fluke South East Asia Pte Ltd., Singapore), calibrated prior to the survey following the manufacturer’s guidelines. Calibration ensured the accuracy and precision of the measurements obtained during the survey. Moreover, all radiation intensities were measured at chest level from 4 positions, at the left lateral, right lateral, anterior, and posterior positions, at distances of 30, 60, 100, and 200 cm postinjection, and at time points of 0 h (injection time), 1.5 h (waiting time), and 3 h (scanning/discharge time), as shown in Figure 1. Ionizing radiation in terms of deep doses was calculated to quantify the overall mean dose rate, and standard deviation was calculated for all time points and distances. This study was approved by the Office of Human Research Ethics Committee, Faculty of Medicine, Prince of Songkla University, Thailand (REC. 64-196-7-2, 24 June 2021).

### 2.2. The ^99m^Tc Radionuclide Decay over Time

To calculate ^99m^Tc radionuclide decay over time, a differential equation was used to calculate the number of radio atoms or the activity (*A*) at a specific time (*t*), as shown in Equation (1):

(1) $$A_{t}=-\frac{{dN}_{t}}{dt}=\lambda \times N_{t}$$

### 2.3. Statistical Analysis

In this study, all experiments were completed in triplicate (experimentally and analytically), and all results and experimental data are shown as mean ± standard deviation. GraphPad Prism 8.0 software (GraphPad Software Inc., Boston, MA, USA) was used for all statistical analyses. One-way ANOVA (“analysis of variance”) was used to compare the means of two or more additional independent groups to ascertain if the statistical evidence suggests that the means are significantly different, with *p*-value < 0.05 considered as being statistically important.

## 3. Results

### 3.1. Mean Radiation Doses at Different Times and Distances on the Left Lateral, Right Lateral, Anterior, and Posterior Positions

This study investigated radiation emitted from the bodies of patients undergoing bone scintigraphy, who were injected with the radiopharmaceutical ^99m^Tc-MDP, to assess potential risks from environmental ionizing radiation. The mean radiation dose measurements were taken at chest level to provide a more accurate radiation dose assessment for the body’s most critical organs. The selection of radiation exposure measurement at the chest level was primarily based on several factors related to human anatomy, radiological protection, and practical considerations. Of note, a study by Günay et al. took measurements from both the head and chest, with the same results for both positions [18]. The radiation intensities emitted from 100 bone scintigraphy patients were quantified, and the data were analyzed in order to assess environmental radiation levels in the vicinity of patients in the postinjection isolation room to determine if levels were within ICRP guidelines. ICRP 105, “Radiation Protection in Medicine”, does not state a medical exposure dose limits to patients, as the radiation dose is specific to the medical condition [4]. However, ICRP 75, “General Principles for the Radiation Protection of Workers”, [5] recommends an effective dose limit of 20 mSv/y (averaged over 5 years) for nuclear medicine staff, and for members of the public, ICRP 60 advises an effective dose limit of 1 mSv/y [19].

Table 1 indicates the mean dose rates recorded at 16 positions around the patient’s body at chest height at three time points, namely 0 h (injection time), 1.5 h (waiting time), and 3 h (scanning/discharge time). The highest mean dose rate of 24.57 µSv/h (range: 6.30–49.00 µSv/h) was recorded at the posterior position at 30 cm and 0 h, and decreased to 8.45 µSv/h after 3 h postinjection, whereas the lowest mean radiation dose at 30 cm, 0 h was 15.07 µSv/h (range: 8.20–27.00 µSv/h), recorded at the right lateral position, and decreased to 4.82 µSv/h after 3 h. The anterior mean dose was 23.35 µSv/h and decreased to 7.56 µSv/h at 3 h. The potential highest risk to medical staff is the posterior position at 0 h, 30 cm, with the highest dose rate of 49.00 µSv/h and 26.80 µSv/h recorded at 60 cm, 1.5 h. This is followed by the anterior position, for which 38.00 µSv/h was recorded at 0 h, 30 cm, and 20.00 µSv/h was recorded at 0 h, 60 cm. In addition, the highest left lateral doses were 44.00 µSv/h, recorded at 0 h, 30 cm, and 38.00 µSv/h at 0 h, 30 cm. The highest right lateral dose was 27 µSv/h, recorded at 0 h, 30 cm. The data indicate that the left and right lateral mean doses were reduced by ~80% as distance increased from 30 to 200 cm, and a reduction of ~67% took place from 0 to 3 h. Similarly, the anterior and posterior mean doses were reduced by ~84% as the distance was increased from 30 to 200 cm, and a ~66% reduction took place from 0 to 3 h.

**Figure 2:**
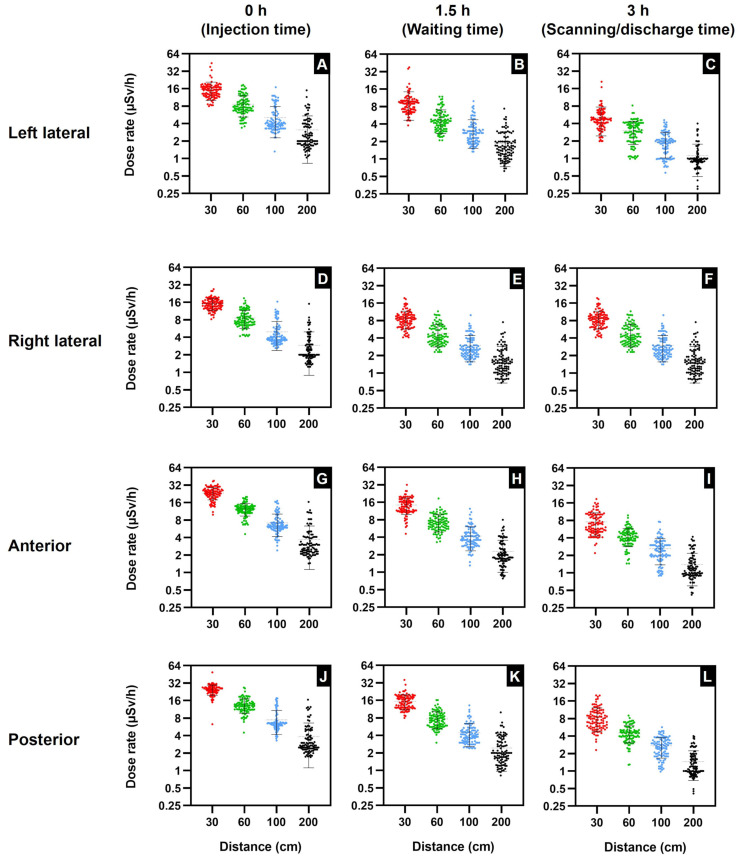
Scatter plot illustrating the data relationship between dose rate (µSv/h) and distance at 30, 60, 100, and 200 cm at 0 h (injection time), 1.5 h (waiting time), and 3 h (scanning/discharge time). Left lateral: (**A**) 0 h, (**B**) 1.5 h, (**C**) 3 h. Right lateral: (**D**) 0 h, (**E**) 1.5 h, (**F**) 3 h. Anterior: (**G**) 0 h, (**H**) 1.5 h, (**I**) 3 h. Posterior: (**J**) 0 h, (**K**) 1.5 h, (**L**) 3 h. Data are shown as total values with mean ± standard deviation (*n* = 100).

In addition, the scatter plots in Figure 2 illustrate the maximum dose in relation to the distance from the patients at various time points. It can be observed that the dose rates in Figure 2A–F are primarily under the 25 µSv/h occupational limit. The exceptions are the left lateral position (Figure 2A) at 0 h and 30 cm, with 4% of the dose readings exceeding the occupational limit, and the left lateral position at 1.5 h and 30 cm, with 2% of the dose readings exceeding the limit. However, the most significant doses exceeding the occupational dose limit were recorded at the anterior and posterior locations. Figure 2G (anterior) and Figure 2J (posterior) illustrate that a substantial proportion of the dose readings exceeds the occupational exposure limit.

The graphs in Table 1 and Figure 2 clearly show the correlation between the mean dose rate and time, and indicate that the mean dose rate at all positions significantly declined from 0 h, the time of intravenous injection, up to 3 h, the time of completion of scintigraphy scans and patients leaving the isolation room. Therefore, the reduction in mean dose rates as time increases indicates that time is also one of the key factors in the mean dose rate’s reduction. Furthermore, the graphs in Figure 2 demonstrate that the position, distance, and time all influenced the emitted external radiation mean dose rate levels. These levels differ as a consequence of ^99m^Tc-MDP’s anatomical distribution in patients’ bodies, time, and distance. The effect of anatomical distribution can be observed in Figure 2, where both the posterior and anterior positions at all distances and time points exhibit higher mean dose rates than either the left lateral or the right lateral positions. As stated earlier, according to Francis et al., 50–60% of ^99m^Tc-HDP diphosphonate tracer bone uptake occurs within 3–4 h [20]. Moreover, Blake et al. studied the protein binding of ^99m^Tc-MDP to determine the in vivo quantity of free ^99m^Tc-MDP as a ratio of renal plasma clearance. They determined that between 2 and 4 h after intravenous injection of ^99m^Tc-MDP, the biological half-life of free ^99m^Tc-MDP in plasma was 92 min, whereas ^99m^Tc bound to MDP had a biological half-life of 540 min [21]. Therefore, we hypothesize that approximately 50% of the 740 MBq is free and unbound with a half-life of 92 min, which influences the mean radiation rate decline. Furthermore, in order to calculate ^99m^Tc radionuclide decay over time, the differential equation calculates the number of radio atoms or the activity (A) at a specific time (t) as shown in Equation (1) [22]. By means of the differential equation, it was calculated that at 1.5 h, the ^99m^Tc activity was 622.34 MBq, and at 3 h, the activity was 523.18 MBq, a reduction of −15.9%. Therefore, ^99m^Tc radionuclide decay over time influences the mean radiation rate decline.

Additionally, patients undergoing bone scintigraphy are encouraged to drink water prior to the scan to encourage the regular voiding of the urinary bladder and increase the rate of free ^99m^Tc-MDP elimination from the body. Research by Blake et al. found that immediately after injection, protein binding of ^99m^Tc-MDP varies from 25 to 30%, increasing to 44–55% at 4 h. Consequently, a substantial proportion of the ^99m^Tc-MDP is unbound during the 3 h of the bone scintigraphy, and the remaining free ^99m^Tc-MDP is cleared from the body by glomerular filtration in the kidneys [21]. Moreover, Table 1 and Figure 2 indicate that distance is the key factor influencing the mean dose rate reduction and has a greater impact than time.

### 3.2. Occupational Radiation Risks from ^99m^Tc-MDP

Consequently, a number of publications have been produced to reduce the risks of ionizing radiation. The International Atomic Energy Agency (IAEA) has three publications: Safety Fundamentals, Safety Requirements, and Safety Guides. These publications promote safety objectives, safety requirements to protect people and the environment, and safety guidelines on fulfilling the requirements [23]. Furthermore, the IAEA, in conjunction with the International Labour Organization (ILO), published an Occupational Radiation Protection Guide (IAEA Safety Standards Series No. GSR Part 3) [24], providing guidance on occupational radiation protection and providing workers with the necessary guidance on monitoring and assessing external radiation exposure from radionuclides. Additionally, the IAEA publication Radiation Protection and Safety in Medical Uses of Ionizing Radiation No. SSG–46 is specifically for patients and nuclear medicine staff, and imparts guidance on minimizing the radiation risks from medical ionizing radiation [25].

Moreover, Figure 2 shows the maximum dose rate declining as a result of increasing the distance and time, with a significant difference at all distances and times (*p* < 0.05). Additionally, the maximum dose data determined that the left lateral maximum doses were reduced by ~60% from 30 to 200 cm and the right lateral were reduced by ~44%. The left lateral maximum dose reduction from 0 to 3 h was ~52%, and the right lateral reduction was ~59%. Furthermore, the anterior maximum dose was reduced by ~57% from 30 to 200 cm, and the reduction from 0 to 3 h was ~51%. Similarly, the posterior maximum dose was reduced by ~66% from 30 to 200 cm, and the reduction from 0 to 3 h was ~59%.

**Figure 3:**
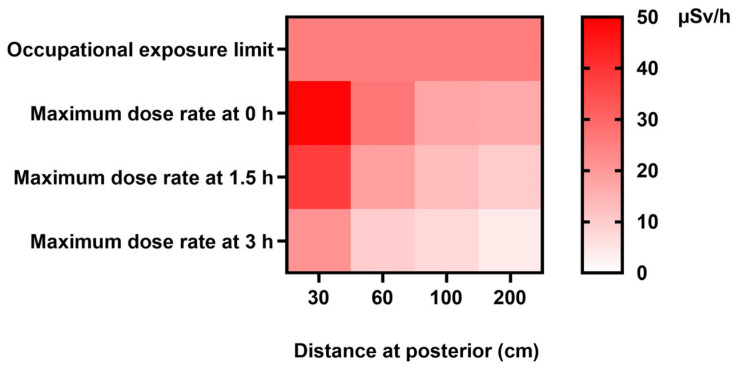
Occupational exposure limit heat map showing maximum dose rates (µSv/h) at 0, 1.5, and 3 h at distances of 30, 60, 100, and 200 cm. Note: According to the International Commission on Radiological Protection (ICRP) Publication 75, titled “General Principles for the Radiation Protection of Workers,” it is recommended that workers be subjected to an effective dose limit of 20 mSv per year, averaged over a period of five consecutive years. Additionally, a maximum limit of 50 mSv per year is advised for any single year. The occupational hourly dose rate of 25 µSv/h is derived using a standard work schedule of 40 h per week, maintained over a span of 50 weeks annually.

Furthermore, the heat map in Figure 3 similarly illustrates the maximum dose rates emitted from patients at various distances and time points, highlighting the distances and times when nuclear medicine staff are potentially at the most significant risk of radiation exposure.

**Figure 4:**
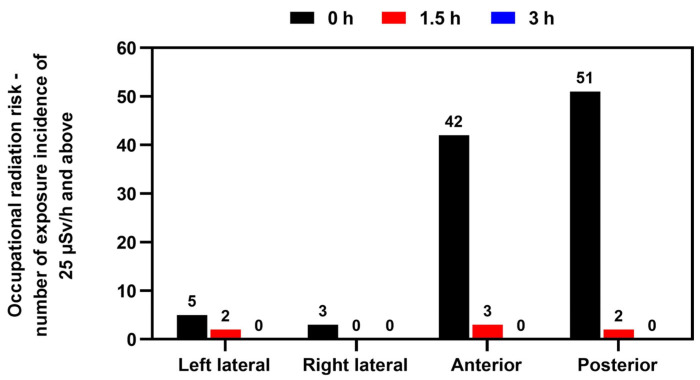
Occupational radiation risk incidence over exposure limit of 25 µSv/h and above at 0 h (time of injection), 1.5 h, and after 3 h on completion of the bone scintigraphy at left lateral, right lateral, anterior, and posterior positions. The International Commission on Radiological Protection (ICRP) 75, “General Principles for the Protection of Workers”, proposes an effective dose limit of 20 mSv/y (averaged over five consecutive years) and a maximum of 50 mSv/y in any single year. Working 40 h per week for 50 weeks per year results in an occupational hourly dose rate of 25 μSv/h. Data are given as total incidences of occupational radiation risk over the exposure limit of 25 µSv/h and above (*n* = 100).

Likewise, Figure 4 further verifies that the greatest risk of radiation exposure to staff was at the anterior and posterior locations. As can be observed, the incidence of doses above the occupational limit dropped significantly after 1.5 h. This study’s findings highlight that regular bone scintigraphy procedures can expose medical staff to radiation levels above the working limits.

### 3.3. Forecasting Dose Rate based on Distance and Time after Injection: Exponential Regression Analysis

Estimating the forecasting radiation dose rate based on distance and time after injection is crucial for radiation exposure risk assessment in various scenarios. Understanding how radiation dose decreases with distance and time allows for a better assessment of potential health risks and aids in developing safety protocols. In medical settings, such as nuclear medicine or radiation therapy, estimating the radiation dose at various distances and times is essential for optimizing treatment plans and minimizing radiation exposure to healthy tissues. By estimating the forecasting radiation dose based on distance and time after injection, risk assessors, health physicists, radiation protection professionals, and medical personnel can make informed decisions, implement appropriate safety measures, and protect individuals and the environment from the potentially harmful effects of radiation exposure. Using reliable models and data in these assessments ensures accuracy and validity [26].

**Figure 5:**
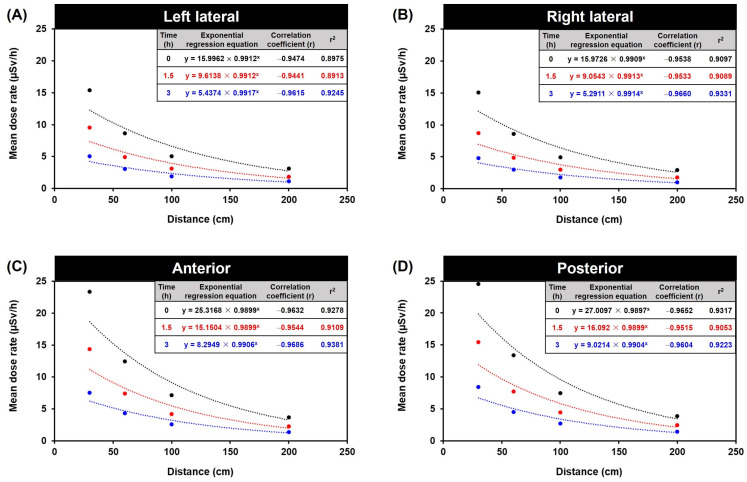
Forecasting dose based on distance and time after injection in exponential regression equation. The mean dose rate relationship between distance and time: (**A**) left lateral, (**B**) right lateral, (**C**) anterior, (**D**) posterior. The exponential regression equation is shown in $y=a\times b^{x}$ where *y* is mean dose rate (μSv/h) and *x* is the distance from the patient (cm).

In exponential regression analysis, the estimated regression coefficients of the time and distance components reflect an exponential regression time trend in the continuous distance response variable. For the statistical data shown in Figure 5 and Table 2, the explanatory variables are distance (distances of 30, 60, 100, and 200 cm; fixed factor for time) and time (0, 1.5, and 3 h; continuous distance). The exponential regression equation is shown in Equation (2):

(2) $$y=a\;\times \;b^{x}$$

where *y* is the mean dose rate (μSv/h) and *x* is the distance from the patient (cm).

Moreover, the exponential regression analysis revealed that the radiation dose substantially affected the distance variable (Figure 5 and Table 2), as the results indicated that the radiation dose decreased proportionally with increasing distance. Additionally, the model considered individual differences in radiation dose, emphasizing the significance of radiation protection in nuclear medicine. The results suggest that the exponential regression analysis approach is useful for comprehending the complex radiation dose relationship between distance and time. Exponential regression analysis is a valuable tool for predicting and gaining insight into the radiation decay patterns of variables.

The dose rate pattern is marked by an initial exponential increase in the dose rate, succeeded by a subsequent exponential decline in the dose rate. As a result, the correlation coefficient (r) is a measure of the strength and direction of the relationship between the forecasting variables (distance and time after injection) and the forecasting variable (dose). The correlation coefficient ranges from −1 to 1, where −1 indicates a perfect negative correlation, 1 indicates a perfect positive correlation, and 0 indicates no correlation [27,28,29]. In addition, the fold change from 0 h represents the ratio of the forecasting dose at a specific time after injection to the forecasting dose at 0 h (immediately after injection). It quantifies the change in dose relative to the initial dose.

**Figure 6:**
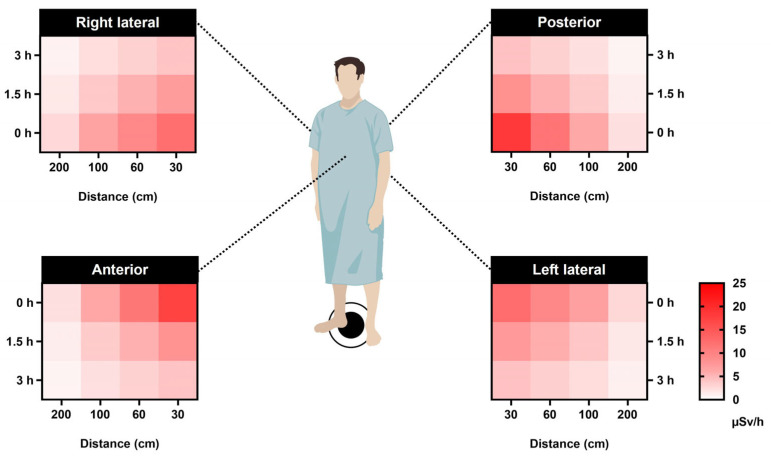
Forecasting dosage based on distance and time after injection presented as an exponential regression heat map. The correlation between distances (30, 60, 100, and 200 cm) at three different time points—0 h (injection time), 1.5 h (waiting time), and 3 h (scanning/discharge time)—for the left lateral, right lateral, anterior, and posterior orientations. The dose calculation was performed using exponential regression formulas derived from the data presented in Figure 5. These formulas take the form of $y=a\times b^{x}$, where *y* represents the average dose rate (μSv/h) and *x* corresponds to the distance from the patient (cm).

As a result, based on the best-fit model parameters for predicting dose rates, we can forecast the radiation responses as a function of time since the beginning of exposure to various time-dependent dose rates. This prediction is made for different distances (30, 60, 100, and 200 cm) at three specific time points: 0 h (time of injection), 1.5 h (waiting period), and 3 h (scanning/discharge time). These forecasts are applicable for the left lateral, right lateral, anterior, and posterior orientations (Figure 6).

### 3.4. Patient Discharge Comparison with Public Exposure Limit from ^99m^Tc-MDP

The establishment of radiation dose limits is intended to safeguard the well-being and security of the broader populace by mitigating the risks associated with potential exposure to ionizing radiation with dose limits for individuals in the general public. The numbers align with established radiation dosage limitations advocated by several international organizations, including the International Commission on Radiological Protection (ICRP) and the US Nuclear Regulatory Commission (NRC). In addition, there is an annual limit that is commonly recommended for radiation exposure among the general population: an annual limit of 0.1 rem (1 mSv) for the total effective dose equivalent (TEDE). As mentioned earlier, the limit encompasses cumulative exposure from many sources of ionizing radiation, including medical operations, naturally occurring background radiation, and radiation originating from human activities. Therefore, the hourly limit in unrestricted areas refers to the maximum number of units or activities that can be performed within a given hour without any restrictions or limitations. The hourly restriction of 0.002 rem (0.02 mSv) from external sources in unrestricted regions pertains to the upper limit of radiation exposure that an individual can sustain within one hour in locations where public access is not limited. As a result, the public exposure limit is 20 µSv/h in unrestricted areas for individual members of the public. This restriction aims to mitigate the risk of humans being subjected to excessive radiation in public areas [30,31,32,33,34].

Moreover, the NRC establishes regulatory guidelines for permissible radiation doses emanating from facilities licensed by the NRC. These guidelines are outlined in Title 10 of the Code of Federal Regulations (CFR), specifically Section 20. The methodology for complying with the dosage limitations specified in 10 CFR 20.1301 is outlined in Section 10 CFR 20.1302. There are two options available for demonstration purposes. The first option involves providing evidence that the annual dose limit is within the maximum. The second option entails demonstrating two conditions: (a) ensuring that the average concentrations of radioactive material released in gaseous and liquid effluents at the boundary of the unrestricted area do not exceed the specified values in Table 2 of Appendix 2 Part 20; and (b) confirming that if an individual was continuously present in an unrestricted area, the dose from external sources would not exceed 0.002 rem (0.02 mSv) in an hour (20 μSv/h) or 0.05 rem (0.5 mSv) in a year [33,35].

**Figure 7:**
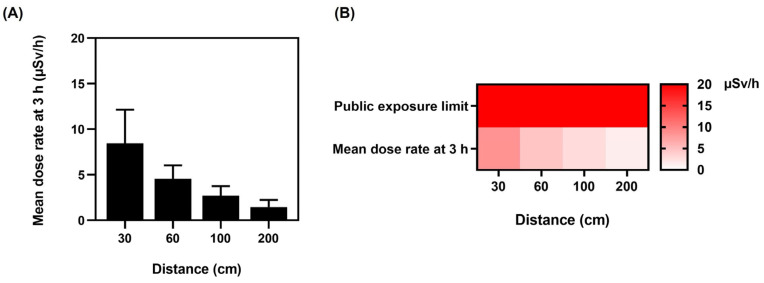
Public exposure limits. (**A**) Mean dose rate at distances of 30, 60, 100, and 200 cm from patient after 3 h on completion of the bone scintigraphy. (**B**) Public exposure limit heat map showing the comparison between mean dose rates at distances of 30, 60, 100, and 200 cm after 3 h on completion of the bone scintigraphy. In an unrestricted area, the United States Nuclear Regulatory Commission (NRC) specifies in Title 10 of the Code of Federal Regulations (CFR) Section 20 that the dose from external sources cannot exceed the reference level of 0.002 rem (0.02 mSv) per hour (20 μSv/h). Data are given as mean ± standard deviation (*n* = 100).

With regard to public safety, after 3 h, on the completion of the bone scintigraphy and the patients’ discharge, all mean dose rates were significantly below the 1 mSv/y public exposure limit based on guidance provided by the Nuclear Regulatory Commission (NRC) Publication Dose Limits [36,37]. On discharge and release to public areas, the patients’ mean dose rates (Figure 7A) were as follows: 1.13 ± 0.64 µSv/h for the left lateral, 1.04 ± 0.53 µSv/h for the right lateral, 1.39 ± 0.64 µSv/h for the anterior, and 1.46 ± 0.77 µSv/h for the posterior.

The heat map (Figure 7B) verifies that dose rates did not exceed the public exposure limit of 20 µSv/h in unrestricted areas for individual members of the public, and Figure 7A indicates that the dose rate at 3 h on completion of the scan was significantly below the public limit. The general public cannot access patients in the postinjection isolation room at our institute, as it is a designated controlled area. Subsequently, on completion of bone scintigraphy, patient discharge is only permitted when radiation levels are substantially lower and within the guidelines.

## 4. Discussion

The gamma radiation from ^99m^Tc-MDP is controlled in order to minimize exposure to the patient and healthcare personnel. Prior to the scan, patients are provided with instructions regarding any precautions related to radiation exposure and contact with others. For a ^99m^Tc-MDP bone scan, the effective dose to the public is generally less than 1 mSv. Bone scintigraphy using the radiotracer ^99m^Tc results in a whole-body effective radiation dose to the patient of 0.0057 mSv per MBq, which is equivalent to around 4 mSv for a 20 mCi (740 MBq) dose of ^99m^Tc -MDP [38]. In comparison, the average annual background radiation dose for an adult is around 3 mSv, and the average annual cosmic radiation dose for aviation personnel is 2.19 mSv/y [39], and below the International Committee for Radiological Protection limit [40]. Moreover, medical professionals take measures to minimize radiation exposure during imaging procedures and follow strict guidelines and safety protocols to ensure the safety of both patients and the public. (i) Consistency and Standardization: Using a standard activity for all patients ensures consistency in imaging protocols. This is important for comparing results across different patients and studies. Standardization helps in establishing reference ranges and in the interpretation of images. (ii) Practicality and Efficiency: Preparing individual doses based on patient weight can be time-consuming and logistically challenging. Standardizing the dose simplifies the process and allows for more efficient workflow in a busy clinical setting. (iii) Minimizing Radiation Exposure Variability: Administering a standard activity to each patient can reduce the variability in radiation exposure between patients. This can help maintain a consistent level of image quality and optimize the trade-off between image quality and radiation dose. For radiological protection, ICRP Publication 103 explains the effective dose and equivalent dosage for organs and tissues in detail. However, UK research comparing nuclear medicine dose estimates for various unintentional exposures indicated significant discrepancies, highlighting the need for a uniform exposure estimation method to eliminate inconsistencies. In addition, because the dangers of low dosages are unknown, a recent Lancet article emphasized the need to adhere to the fundamental principles of radiological protection, and scanning procedures should use the lowest feasible dosage. Moreover, the study found a robust dose–response association between brain radiation dosage and the relative risk of all brain malignancies [41].

Despite increased concern about radiation exposure to medical staff, a review of South Korean medical staff’s lifetime cancer risk found that the predicted risk was modest in most situations [42]. Despite this, radiological technicians had the greatest cancer rate, followed by nurses. Although female workers were exposed to lower radiation levels than male workers, radiation was expected to increase their cancer risk, and both male and female staff had a greater risk of bladder and thyroid cancer. Although the occupational risk of exposure to ionizing radiation remains a concern for medical staff, the downward trend is encouraging. Therefore, continuous monitoring is necessary to prevent future potential problems [43]. Occupation-related radiation-induced cataracts are a recent issue [44], with gender, age, and heredity playing crucial roles [45]. Therefore, in light of our findings and the high dose rates recorded, we recommend that staff wear adequate eye protection in the vicinity of patients undergoing bone scans. In the rapidly expanding field of medical imaging and image-guided therapy, medical personnel must be made aware of potential radiation concerns to reduce radiation exposure, and must continually reinforce the basics of radiation protection. This research highlights the radiation hotspots during bone scans to enable staff to be more vigilant.

The mean dose in this study at 0 h, 30 cm at all positions was 19.60 µSv/h (Table 1), similar to the results of Fathy et al., who reported a mean dose of 23.60 µSv/h [46]. Additionally, Al-Esaei et al. determined that the mean radiation dose for ^99m^Tc-MDP bone scans for medical staff was 0.527 μSv and the annual effective dose was 1.34 mSv [47]. Although the mean dose rates at our center were below the limit, we established that the critical time and distance when medical staff are most at risk from radiation exposure is immediately after the ^99m^Tc intravenous injection (Figure 2), when dose levels exceed the occupational limit. The highest dose rates were recorded from 51% of the patients, emitting ≥25 µSv/h up to 49.00 µSv/h at the posterior position at a distance of 30 cm. Additionally, at the anterior location at a distance of 30 cm, 42% of patients emitted ≥25 µSv/h up to 38.00 µSv/h. Furthermore, at 1.5 h after the tracer injection, 7% of the radiation dose rates exceeded 25 µSv/h (Figure 4).

Moreover, it was ascertained that dose levels differed as a consequence of ^99m^Tc-MDP’s biological distribution in patients’ bodies, time, and distance from the source, as illustrated in Figure 2, which indicates that anatomical location, distance, and time influence the radiation dose rate levels. The effect of biological distribution and anatomical location can be observed in Figure 2, where both the posterior and anterior locations at all distances and time points exhibit higher mean dose rates than either the left or the right lateral positions. ^99m^Tc-MDP is retained in the kidney and urinary bladder, skeletal uptake is uniform [48], and 2–10% is usually distributed in soft tissue [49]. Furthermore, the relationship between the mean dose rate and the time points indicates that the mean dose rate decreased as time increased. According to Francis et al., 50–60% of ^99m^Tc-HDP diphosphonate uptake occurs within 3–4 h [20]. Moreover, Blake et al. found that immediately after injection, the protein binding of ^99m^Tc-MDP varies from 25 to 30%, increasing to 44–55% at 4 h. It was verified that 2–4 h after the intravenous injection of ^99m^Tc-MDP, the biological half-life of free ^99m^Tc-MDP in plasma was 92 min, whereas ^99m^Tc bound to MDP had a biological half-life of 540 min [21]. Consequently, a substantial proportion of the ^99m^Tc-MDP is unbound during the 3 h of the bone scintigraphy, and the remaining free ^99m^Tc-MDP is cleared from the body via glomerular filtration in the kidneys. Therefore, we hypothesize that approximately 50% of the 740 MBq is free and unbound with a half-life of 92 min, influencing the radiation dose rate decline. Furthermore, in order to calculate ^99m^Tc radionuclide decay over time, a differential equation was used to calculate the number of radio atoms or the activity (A) at a specific time (t), as shown in Equation (1). By means of the differential equation [22], it was calculated that at 1.5 h, the ^99m^Tc activity was 622.34 MBq, and at 3 h, the activity was 523.18 MBq, resulting in a reduction of −15.9%. Therefore, ^99m^Tc radionuclide decay over time influences the dose rate decline. However, this study identified that the greatest dose reduction was the result of increasing the distance from the radiation source. This is a consequence of the inverse square law, which states that the amount of radiation received decreases the farther the radiation travels by a factor of 1 over the distance squared [50].

In addition, the statistical mean is often used to calculate the central tendency of data in order to create a statistical summary. However, the arithmetic mean can provide a misleading perspective and a false sense of safety when extreme numbers occur. Furthermore, more than simply observing the arithmetic mean is required to enable an accurate conclusion about the data in this study, as a significant number of doses recorded at several positions and time points exceeded the occupational exposure limit. To determine the relationship between radiation dose, distance, and time, exponential regression analysis enabled the forecasting of radiation doses from bone scans after injection at different distances and exposure times (Table 2). This verified that the radiation dose had a significant effect on the distance variable, and the radiation dose dropped proportionally with increasing distance.

To reduce the risks from radiation, a number of publications have been produced. The International Atomic Energy Agency (IAEA) has three publications: Safety Fundamentals [51], Safety Requirements, and Safety Guides [23]. Furthermore, the IAEA, in conjunction with the International Labour Organization (ILO), published an Occupational Radiation Protection Guide (IAEA Safety Standards Series No. GSR Part 3) [24]. Additionally, the IAEA publication Radiation Protection and Safety in Medical Uses of Ionizing Radiation No. SSG–46 is specifically for patients and nuclear medicine staff, imparting guidance on minimizing the radiation risks from medical ionizing radiation [25]. “Dosage constraints and reference levels in radiological protection”, ICRP Publication 103, lists effective doses for organs and tissues. However, twenty-seven hospital radiopharmacies estimated the exposure in various accidental exposure scenarios, resulting in a substantial variation in dose estimates [52]. Notably, the results emphasized the importance of implementing a standard method to estimate exposure to avoid inconsistencies.

Moreover, because the dangers of low radiation dosages are unknown, a recent Lancet article emphasized the need to adhere to the fundamental principles of radiological protection and justify diagnostic treatments employing ionizing radiation [41], stating that scans should use the lowest feasible dosage. Additionally, the study found a robust dose–response association between brain radiation dosage and the relative risk of all brain malignancies, stating that 5–15 years after a single head CT examination (with an average brain dose of 38 mSv), approximately one case of radiation-induced brain cancer is expected for every 10,000 people. Additionally, a recent study by Little et al. supports a link between acute high-dose and chronic low-dose radiation exposure and cardiovascular disease, stating that low dosage and low dose rate exposure are associated with a greater risk per unit dose [53]. In addition, patient communications are important, as Ribeiro et al. studied the ^99m^Tc-MDP bone scan radiation exposure awareness of patients undergoing scans, and identified a lack of awareness about ionizing radiation exposure and bone scan radiation levels [54]. Furthermore, a poll at a hospital determined medical staff required more training, particularly regarding radiation-related illnesses [55].

Moreover, this study ascertained that the risk to the public from patients discharged after bone scintigraphy was within the current guidelines, and the mean radiation doses from ^99m^Tc-MDP were below occupational limits. However, a potential risk to medical staff is associated with bone scintigraphy. This study highlights the potential risk to staff in the early stages of bone scintigraphy, so all staff must be informed of the potential risks, and management must ensure all staff have regular safety training, wear adequate protection, maintain a safe distance when in the vicinity of patients, and limit their exposure time [13].

## 5. Conclusions

The results conclude that the mean radiation levels decreased with increased distance and time (*p*-value < 0.05) due to gamma radiation decay, biological clearance, and increased distance. As a result, it was determined that the mean radiation dose from patients on discharge was below the public exposure limit for external radiation. Additionally, the exposure for the nuclear medicine staff was less than the recommended radiation dose of 25 µSv/h. Nevertheless, although the skeleton absorbs 50% of the injected activity, there was a substantial risk to staff post-intravenous injection. Furthermore, in the exponential regression equation, the anticipated dosage based on distance and time after injection determines the radiation exposure risk assessment in various scenarios. It indicates the relationship between distance at 30, 60, 100, and 200 cm at 0 h (injection time), 1.5 h (waiting time), and 3 h (scanning/discharge time) and the left, right, anterior, and posterior positions. As a result, it was observed that when the distance from the patient increased for all positions, there was a decrease in statistical significance, which was confirmed by conducting an exponential regression analysis. Additionally, after the bone scintigraphy was completed, the patients’ dose rates on discharge were within the current guidelines, and the mean radiation doses from ^99m^Tc-MDP were below occupational limits. Therefore, staff require regular training to understand the risks and adhere to correct working practices. On discharge and release to public areas, the patients’ mean dose rates were as follows: 1.13 µSv/h for the left lateral, 1.04 µSv/h for the right lateral, 1.39 µSv/h for the anterior, and 1.46 µSv/h for the posterior. Furthermore, while this study focused on radiation risks to nuclear medicine workers, it is critical that all patients’ cumulative radiation exposure levels are included in a patient dose management system and that cumulative radiation exposure notifications are provided.

## Figures and Tables

**Table 1:** Mean radiation dose ± standard deviation (range: minimum–maximum dose) (µSv/h) at distances of 30, 60, 100, and 200 cm at 0 h (injection time), 1.5 h (waiting time), and 3 h (scanning/discharge time) on the left lateral, right lateral, anterior, and posterior positions.

Patient Position	Distance from Patient (cm)	Time after the Injection (h)		
		0	1.5	3
		Mean Dose Rate ± SD (Range) µSv/h	Mean Dose Rate ± SD (Range) µSv/h	Mean Dose Rate ± SD (Range) µSv/h
Left lateral	30	15.43 ± 5.41 (8.00–44.00)	9.53 ± 4.87 (3.80–38.00)	5.09 ± 2.64 (1.99–21.00)
	60	8.65 ± 3.44 (3.40–18.60)	4.90 ± 2.14 (2.10–11.90)	3.06 ± 1.32 (0.99–8.20)
	100	5.09 ± 2.81 (1.32–17.00)	3.13 ± 1.61 (1.34–9.90)	1.91 ± 0.89 (0.57–4.60)
	200	3.12 ± 2.30 (1.04–15.00)	1.85 ± 1.12 (0.62–7.40)	1.13 ± 0.64 (0.30–4.00)
Right lateral	30	15.07 ± 3.43 (8.20–27.00)	8.69 ± 2.99 (4.10–19.60)	4.82 ± 2.08 (1.90–11.00)
	60	8.60 ± 2.96 (4.20–18.80)	4.84 ± 2.03 (2.30–11.60)	3.02 ± 1.38 (0.93–9.20)
	100	4.94 ± 2.58 (2.60–16.50)	3.00 ± 1.43 (1.42–9.90)	1.79 ± 0.79 (0.72–3.90)
	200	2.92 ± 2.02 (1.25–15.10)	1.77 ± 1.09 (0.68–7.50)	1.04 ± 0.53 (0.34–3.00)
Anterior	30	23.35 ± 5.49 (9.90–38.00)	14.36 ± 4.55 (4.60–32.00)	7.56 ± 3.23 (2.20–18.70)
	60	12.44 ± 3.04 (4.60–20.00)	7.39 ± 2.46 (3.30–18.60)	4.34 ± 1.51 (1.45–9.80)
	100	7.18 ± 3.02 (2.40–17.20)	4.20 ± 1.87 (1.31–12.40)	2.62 ± 1.25 (0.89–7.60)
	200	3.72 ± 2.60 (1.43–16.50)	2.28 ± 1.28 (0.78–8.00)	1.39 ± 0.79 (0.42–4.20)
Posterior	30	24.57 ± 5.03 (6.30–49.00)	15.47 ± 4.49 (8.00–36.00)	8.45 ± 3.68 (2.30–20.00)
	60	13.37 ± 3.73 (4.50–26.80)	7.71 ± 2.49 (3.00–16.10)	4.54 ± 1.48 (1.28–9.00)
	100	7.50 ± 3.31 (3.30–17.60)	4.47 ± 1.90 (2.40–13.10)	2.71 ± 1.04 (0.98–5.70)
	200	3.85 ± 2.74 (1.70–16.60)	2.43 ± 1.47 (0.82–9.90)	1.46 ± 0.77 (0.41–4.00)

**Table 2:** Forecasting dose based on distance and time after injection in exponential regression equation. The relationship between distance at 30, 60, 100, and 200 cm at 0 h (injection time), 1.5 h (waiting time), and 3 h (scanning/discharge time) at left lateral, right lateral, anterior, and posterior orientations.

Patient Position	Distance (cm)			Time (h)			
		0		1.5		3	
		(Exponential Regression) Dose * (μSv/h)	Fold Change from 0 h at 30 cm	(Exponential Regression) Dose * (μSv/h)	Fold Change from 1.5 h at 30 cm	(Exponential Regression) Dose * (μSv/h)	Fold Change from 3 h at 30 cm
Left lateral	30	12.27		7.37		4.23	
	60	9.41	−0.23	5.66	−0.23	3.30	−0.22
	100	6.61	−0.46	3.97	−0.46	2.36	−0.44
	200	2.73	−0.78	1.64	−0.78	1.03	−0.76
Right lateral	30	12.14		6.97		4.08	
	60	9.23	−0.24	5.36	−0.23	3.15	−0.23
	100	6.40	−0.47	3.78	−0.46	2.23	−0.45
	200	2.57	−0.79	1.58	−0.77	0.94	−0.77
Anterior	30	18.67		11.17		6.25	
	60	13.77	−0.26	8.24	−0.26	4.71	−0.25
	100	9.17	−0.51	5.49	−0.51	3.23	−0.48
	200	3.32	−0.82	1.99	−0.82	1.25	−0.80
Posterior	30	19.80		11.87		6.75	
	60	14.51	−0.27	8.75	−0.26	5.06	−0.25
	100	9.59	−0.52	5.83	−0.51	3.44	−0.49
	200	3.41	−0.83	2.11	−0.82	1.31	−0.81

* The (exponential regression) dose was calculated using the exponential regression equations from Figure 5 shown in $y=a\times b^{x}$, where *y* is the mean dose rate (μSv/h) and *x* is the distance from the patient (cm).

## Data Availability

Access to detailed individual data is restricted due to both ethical and legal concerns. Data requests should be reviewed with the corresponding author and approved by the Office of Human Research Ethics Committee, Faculty of Medicine, Prince of Songkla University.

## References

[1] Brambilla M., Vassileva J., Kuchcinska A., Rehani M.M. (2020). Multinational Data on Cumulative Radiation Exposure of Patients from Recurrent Radiological Procedures: Call for Action. Eur. Radiol..

[2] Vassileva J., Holmberg O. (2021). Radiation Protection Perspective to Recurrent Medical Imaging: What Is Known and What More Is Needed?. Br. J. Radiol..

[3] Rehani M.M., Yang K., Melick E.R., Heil J., Šalát D., Sensakovic W.F., Liu B. (2020). Patients Undergoing Recurrent CT Scans: Assessing the Magnitude. Eur. Radiol..

[4] Protection R. (2007). ICRP Publication 103. Ann. ICRP.

[5] International Commission on Radiological Protection (1997). General Principles for the Radiation Protection of Workers: Adopted by the Commission in January 1997.

[6] Khamtuikrua C., Suksompong S. (2020). Awareness about Radiation Hazards and Knowledge about Radiation Protection among Healthcare Personnel: A Quaternary Care Academic Center–Based Study. SAGE Open Med..

[7] Love C., Din A.S., Tomas M.B., Kalapparambath T.P., Palestro C.J. (2003). Radionuclide Bone Imaging: An Illustrative Review. Radiographics.

[8] Tabakov S., Milano F., Stoeva M.S., Sprawls P., Tipnis S., Underwood T. (2021). Encyclopaedia of Medical Physics: Two Volume Set.

[9] Taylor A.T. (2014). Radionuclides in Nephrourology, Part 1: Radiopharmaceuticals, Quality Control, and Quantitative Indices. J. Nucl. Med..

[10] Orsini F., Puta E., Lorenzoni A., Erba P., Mariani G. (2017). Single-Photon-Emitting Radiopharmaceuticals for Diagnostic Applications. Nuclear Oncology: From Pathophysiology to Clinical Applications.

[11] Shulkin B.L., Mandell G.A., Cooper J.A., Leonard J.C., Majd M., Parisi M.T., Sfakianakis G.N., Balon H.R., Donohoe K.J. (2008). Procedure Guideline for Diuretic Renography in Children 3.0. J. Nucl. Med. Technol..

[12] Stagi S., Cavalli L., Iurato C., Seminara S., Brandi M.L., de Martino M. (2013). Bone Metabolism in Children and Adolescents: Main Characteristics of the Determinants of Peak Bone Mass. Clin. Cases Miner. Bone Metab..

[13] Van den Wyngaert T., Strobel K., Kampen W., Kuwert T., Van der Bruggen W., Mohan H., Gnanasegaran G., Delgado-Bolton R., Weber W., Beheshti M. (2016). The EANM Practice Guidelines for Bone Scintigraphy. Eur. J. Nucl. Med. Mol. Imaging.

[14] Mattsson S., Johansson L., Leide Svegborn S., Liniecki J., Noßke D., Riklund K., Stabin M., Taylor D., Bolch W., Carlsson S. (2015). ICRP Publication 128: Radiation Dose to Patients from Radiopharmaceuticals: A Compendium of Current Information Related to Frequently Used Substances. Ann. ICRP.

[15] Mattar E.H. (2022). Assessment of Patient and Staff Annual Effective Doses at a Nuclear Medicine Department during Bone Scans. Open J. Radiol..

[16] Larkin A.M., Serulle Y., Wagner S., Noz M.E., Friedman K. (2011). Quantifying the Increase in Radiation Exposure Associated with SPECT/CT Compared to SPECT Alone for Routine Nuclear Medicine Examinations. Int. J. Mol. Imaging.

[17] Mettler F.A., Huda W., Yoshizumi T.T., Mahesh M. (2008). Effective Doses in Radiology and Diagnostic Nuclear Medicine: A Catalog. Radiology.

[18] Günay O., Sarihan M., Abamor E., Yarar O. (2019). Environmental Radiation Doses from Patients Undergoing Tc-99m DMSA Cortical Renal Scintigraphy. Int. J. Comput. Exp. Sci. Eng. (IJCESEN).

[19] Eckerman K., Harrison J., Menzel H., Clement C. (2012). ICRP Publication 119: Compendium of Dose Coefficients Based on ICRP Publication 60. Ann. ICRP.

[20] Francis M., Fogelman I. (1987). 99m Tc Diphosphonate Uptake Mechanism on Bone. Bone Scanning in Clinical Practice.

[21] Blake G., Moore A., Park-Holohan S.-J., Fogelman I. (2003). A Directin Vivomeasurement of 99mTc-Methylene Diphosphonate Protein Binding. Nucl. Med. Commun..

[22] Bailey D.L., Humm J. (2014). Nuclear Medicine Physics: A Handbook for Teachers and Students.

[23] World Health Organization (1994). International Basic Safety Standards for Protection against Ionizing Radiation and for the Safety of Radiation Sources.

[24] Safety, IAEA Occupational Radiation Protection Program (2018). Guide GSG-7.

[25] Guide S.S. (2018). Radiation Protection and Safety in Medical Uses of Ionizing Radiation.

[26] Moore Jr R.M., Kaczmarek R.G. (1990). Occupational Hazards to Health Care Workers: Diverse, Ill-Defined, and Not Fully Appreciated. Am. J. Infect. Control.

[27] Asuero A.G., Sayago A., González A. (2006). The Correlation Coefficient: An Overview. Crit. Rev. Anal. Chem..

[28] Taylor R. (1990). Interpretation of the Correlation Coefficient: A Basic Review. J. Diagn. Med. Sonogr..

[29] Weisstein E.W. (2006). Correlation Coefficient. https://mathworld.wolfram.com/.

[30] Carey J.E., Kumpuris T.M., Wrobel M.C. (1995). Release of Patients Containing Therapeutic Dosages of Iodine-131 from Hospitals. J. Nucl. Med. Technol..

[31] Chen M.Y. (2014). Radiation Protection and Regulations for the Nuclear Medicine Physician.

[32] Kocher D. (1987). Environmental Radiation Standards.

[33] Soelberg N.R., Garn T.G., Greenhalgh M.R., Law J.D., Jubin R., Strachan D.M., Thallapally P.K. (2013). Radioactive Iodine and Krypton Control for Nuclear Fuel Reprocessing Facilities. Sci. Technol. Nucl. Install..

[34] Jubin R.T., Soelberg N.R., Strachan D.M., Ilas G. (2012). Fuel Age Impacts on Gaseous Fission Product Capture during Separations.

[35] Cool D., Peterson H. (1991). Standards for Protection against Radiation, 10 CFR Part 20.

[36] Jones C.G. (2019). The US Nuclear Regulatory Commission Radiation Protection Policy and Opportunities for the Future. J. Radiol. Prot..

[37] Klemic G. (1996). Environmental Radiation Monitoring in the Context of Regulations on Dose Limits to the Public.

[38] Grant F.D., Fahey F.H., Packard A.B., Davis R.T., Alavi A., Treves S.T. (2008). Skeletal PET with 18F-Fluoride: Applying New Technology to an Old Tracer. J. Nucl. Med..

[39] Feng Y., Chen W., Sun T., Duan S., Jia B., Zhang H. (2002). Estimated Cosmic Radiation Doses for Flight Personnel. Hang Tian Yi Xue Yu Yi Xue Gong Cheng = Space Med. Med. Eng..

[40] Harrison J., Balonov M., Bochud F., Martin C., Menzel H., Ortiz-Lopez P., Smith-Bindman R., Simmonds J., Wakeford R. (2021). ICRP Publication 147: Use of Dose Quantities in Radiological Protection. Ann. ICRP.

[41] Hauptmann M., Byrnes G., Cardis E., Bernier M.-O., Blettner M., Dabin J., Engels H., Istad T.S., Johansen C., Kaijser M. (2023). Brain Cancer after Radiation Exposure from CT Examinations of Children and Young Adults: Results from the EPI-CT Cohort Study. Lancet Oncol..

[42] Lee W.J., Choi Y., Ko S., Cha E.S., Kim J., Kim Y.M., Kong K.A., Seo S., Bang Y.J., Ha Y.W. (2018). Projected Lifetime Cancer Risks from Occupational Radiation Exposure among Diagnostic Medical Radiation Workers in South Korea. BMC Cancer.

[43] Sharkey A.R., Gambhir P., Saraskani S., Walker R., Hajilou A., Bassett P., Sandhu N., Croasdale P., Honey I., Diamantopoulos A. (2021). Occupational Radiation Exposure in Doctors: An Analysis of Exposure Rates over 25 Years. Br. J. Radiol..

[44] Khan D.Z., Lacasse M.C., Khan R., Murphy K.J. (2017). Radiation Cataractogenesis: The Progression of Our Understanding and Its Clinical Consequences. J. Vasc. Interv. Radiol..

[45] Barnard S.G., Hamada N. (2022). Individual Response of the Ocular Lens to Ionizing Radiation. Int. J. Radiat. Biol..

[46] Fathy M., Khalil M.M., Elshemey W.M., Mohamed H.S. (2019). Occupational Radiation Dose to Nuclear Medicine Staff Due to Tc99m, F18-FDG PET and Therapeutic I-131 Based Examinations. Radiat. Prot. Dosim..

[47] Al-Esaei A.M., Khalil M.M., El Shazly R.M., Kany A.M., Saleh E.E., Elmaghraby S. (2022). Assessment of Radiation Exposure Dose for Nuclear Medicine Workers from 18F-FDG, 99mTc MDP, and 99mTc. Curr. Radiopharm..

[48] Zuckier L.S., Martineau P. (2015). Altered Biodistribution of Radiopharmaceuticals Used in Bone Scintigraphy.

[49] Peller P., Ho V.B., Kransdorf M. (1993). Extraosseous Tc-99m MDP Uptake: A Pathophysiologic Approach. Radiographics.

[50] Bolus N.E. (2008). Review of Common Occupational Hazards and Safety Concerns for Nuclear Medicine Technologists. J. Nucl. Med. Technol..

[51] Aro I. (2005). IAEA Safety Fundamentals: The Safety of Nuclear Installations and the Defence in Depth Concept.

[52] Murray A., Memmott M. (2023). UK Audit of Variation in Nuclear Medicine Occupational Exposure Calculations in 2021. J. Radiol. Prot..

[53] Little M.P., Azizova T.V., Richardson D.B., Tapio S., Bernier M.-O., Kreuzer M., Cucinotta F.A., Bazyka D., Chumak V., Ivanov V.K. (2023). Ionising Radiation and Cardiovascular Disease: Systematic Review and Meta-Analysis. BMJ.

[54] Ribeiro A.S., Husson O., Drey N., Murray I., May K., Thurston J., Oyen W.J. (2020). Radiation Exposure Awareness from Patients Undergoing Nuclear Medicine Diagnostic 99mTc-MDP Bone Scans and 2-Deoxy-2-(18F) Fluoro-D-Glucose PET/Computed Tomography Scans. Nucl. Med. Commun..

[55] Faggioni L., Paolicchi F., Bastiani L., Guido D., Caramella D. (2017). Awareness of radiation protection and dose levels of imaging procedures among medical students, radiography students, and radiology residents at an academic hospital: Results of a comprehensive survey. Eur. J. Radiol..

